# Phenotypic Antibiotic Resistance Patterns of *Escherichia coli* Isolates from Clinical UTI Samples and Municipal Wastewater in a Grenadian Community

**DOI:** 10.3390/ijerph22010097

**Published:** 2025-01-12

**Authors:** Makeda Matthew-Bernard, Karla Farmer-Diaz, Grace Dolphin-Bond, Vanessa Matthew-Belmar, Sonia Cheetham, Kerry Mitchell, Calum N. L. Macpherson, Maria E. Ramos-Nino

**Affiliations:** 1 Department of Microbiology, Immunology, and Pharmacology, School of Medicine, St. George’s University, St. George’s P.O. Box 7, Grenada; mmatthew@sgu.edu (M.M.-B.); kfarmer1@sgu.edu (K.F.-D.); gdolphin@sgu.edu (G.D.-B.); 2 Department of Pathobiology, School of Veterinary Medicine, St. George’s University, St. George’s P.O. Box 7, Grenada; vmatthew@sgu.edu (V.M.-B.); scheetha@sgu.edu (S.C.); 3 Department of Public Health and Preventive Medicine, School of Medicine, St. George’s University, St. George’s P.O. Box 7, Grenada; kmitche3@sgu.edu; 4 School of Graduate Studies, St. George’s University, St. George’s P.O. Box 7, Grenada; cmacpherson@sgu.edu

**Keywords:** antibiotic resistance, phenotypic analysis, clinical isolates, wastewater surveillance, *Escherichia coli*, Grenada

## Abstract

Antimicrobial resistance (AMR) is a growing global health threat. This study investigated antibiotic resistance in *E. coli* isolates from municipal wastewater (86 isolates) and clinical urinary tract infection (UTI) cases (34 isolates) in a Grenadian community, using data from January 2022 to October 2023. Antibiogram data, assessed per WHO guidelines for Critically Important antimicrobials (CIA), showed the highest resistance levels in both clinical and wastewater samples for ampicillin, followed by amoxicillin/clavulanic acid and nalidixic acid, all classified as Critically Important. Similar resistance was observed for sulfamethoxazole-trimethoprim (highly important) in both groups, with nitrofurantoin showing resistance in the important category. According to the WHO AWaRe classification, ampicillin (ACCESS group) had the highest resistance, while nitrofurantoin had the lowest across all samples. The WATCH group antibiotics, cefuroxime and cefoxitin, showed comparable resistance levels, whereas aztreonam from the RESERVE group (tested only in wastewater) was 100% sensitive. Multiple Antibiotic Resistance (MAR) index analysis revealed that 7% of wastewater and 38.2% of clinical samples had MAR values over 0.2, indicating prior antibiotic exposure in clinical isolates. These parallel patterns in wastewater and clinical samples highlight wastewater monitoring as a valuable tool for AMR surveillance, supporting antibiotic stewardship through ongoing environmental and clinical assessment.

## 1. Introduction

Antimicrobial resistance (AMR) is a top 10 global health threat [1,2,3] and a “silent pandemic” [4] projected to be the leading cause of death by 2050 due to rising antibiotic resistance and limited new treatments [1,5,6]. AMR was estimated to be directly responsible for 1.27 million deaths globally in 2019 and contributed to 4.95 million deaths in total that year [4]. When narrowed down to the Latin America and Caribbean region, AMR contributed to 322,000 deaths in 2021, and this number is projected to increase to 650,000 by 2050, resulting in Latin America and the Caribbean accounting for one of the highest AMR mortality rates across all ages [7].

AMR is a complex multiprong issue, including increased global travel, changing trade patterns, climate change, and population growth [8]. Human activities significantly impact this crisis, with the overuse and misuse of antibiotics, misdiagnoses, and the prophylactic use of antibiotics in animal farming all fueling the rise in resistance [5,9,10]. The continued rise of AMR is eroding the efficacy of antibiotics, posing a critical risk to managing widespread bacterial infections and ultimately threatening global public health [11].

Managing antibiotic resistance requires a multifaceted approach, including implementing an empirical treatment strategy supported by ongoing surveillance to monitor resistance patterns and trends. Access to high-quality data that accurately describes the global spread and characteristics of AMR is essential for making informed public health decisions and guiding treatment protocols. This effort requires robust support both nationally and globally to ensure the effective enforcement of public health measures and the appropriate selection of treatments [8]. However, decision-making is often hindered by gaps in patient-specific data, which may be incomplete or unavailable [12]. Additionally, inconsistencies in AMR trend reporting arise because hospitals may follow varied reporting and classification systems, complicating data comparability across regions [10]. This is no different for the Caribbean region, as the true scope of AMR is poorly documented, which can be attributed to a lack of AMR information at the local level and, in some instances, limited laboratory capacity for antimicrobial susceptibility testing [13].

Current AMR surveillance primarily targets a limited range of pathogens and relies on passive reporting of phenotypic lab results from specific pathogens linked to human infections [8]. The COVID-19 pandemic highlighted AMR issues, as the increased use of antimicrobials led to the selection and spread of resistant bacteria and other microorganisms within the environment [14]. Given the challenges of comprehensive AMR monitoring, wastewater surveillance offers a valuable solution, providing a broader view of AMR trends within communities and bridging critical gaps in traditional surveillance methods [12,15].

Urban wastewater offers a thorough, unbiased snapshot of public health for an entire area connected to the sewage system [16]. Untreated wastewater is a complex sample, containing a mix of bodily fluids such as urine and feces, along with household wastewater, making it a rich source of biomarkers that can inform public health monitoring [17]. Unlike clinical testing, which typically tracks individual cases, wastewater-based surveillance provides a community-wide sample that reflects the collective health status of the population served by the wastewater network [15,18,19].

As such, wastewater-based epidemiology (WBE) has emerged as a powerful tool for monitoring antimicrobial resistance (AMR) at the population level, particularly in communities with limited clinical testing capacity [8,15,20].

A significant public health concern related to antimicrobial resistance (AMR) is the rising prevalence of bacteria that are resistant to multiple antibiotics used for treating bacterial infections [5,21]. In the United States, multidrug-resistant infections are alarmingly common, with around three million cases reported, especially in urinary tract infections (UTIs) where standard antibiotics are losing effectiveness [5]. Globally, UTIs affect 150 million people each year [22].

The economic impact of UTI-related symptoms is significant, leading to over ten million office visits and approximately three million emergency department visits annually, with associated costs reaching billions [5,23]. Among the bacterial pathogens causing UTIs, *Escherichia coli*—particularly uropathogenic *E. coli* (UPEC)—is one of the most common [24].

**Figure 1:**
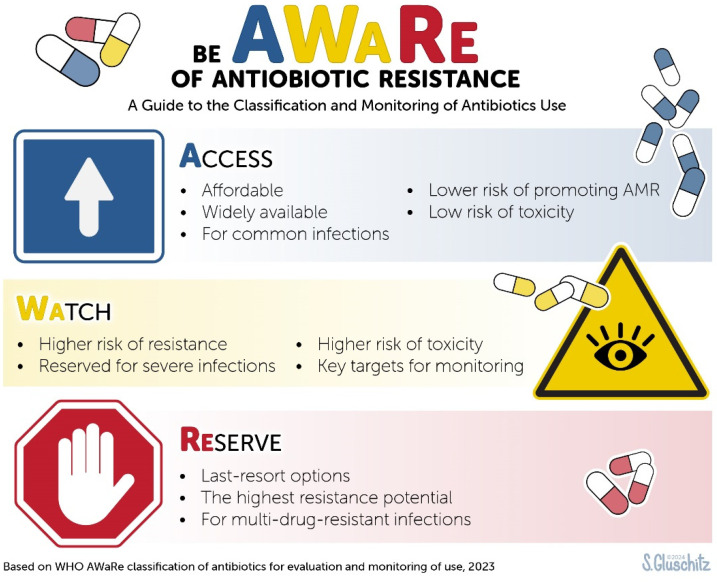
WHO AWaRe classification.

The World Health Organization’s (WHO) AWaRe classification [25,26] (Figure 1) and the WHO Critically Important Antimicrobials for Human Medicine list (CIA) [26] are key tools for managing antimicrobial use and assessing resistance risks globally. The AWaRe classification categorizes antibiotics into three groups as follows: ACCESS (first-line treatments with low resistance potential), WATCH (antibiotics with higher resistance risk, to be used sparingly), and RESERVE (last-resort antibiotics for severe infections). This framework advocates for the use of amoxicillin/clavulanic acid, nitrofurantoin, and sulfamethoxazole/trimethoprim (or trimethoprim alone) for treating acute lower UTIs [25,26]. The CIA categorizes antibiotics into three groups based on their importance for treating severe human infections and their potential to drive antimicrobial resistance (AMR): (1) critical antibiotics are essential for life-threatening infections, with limited or no alternatives, and should be reserved for the most urgent cases to prevent resistance, (2) important antibiotics are used for a broad range of infections where alternatives exist and should be used carefully to minimize resistance, and (3) highly important antibiotics are also crucial, but they have more available alternatives, making them less critical than the other two groups. The list includes antibiotics used exclusively in humans, those used in both humans and animals, and those used only in animals but still posing a risk to human health through transmission or environmental exposure.

The MAR index complements these frameworks by tracking resistance patterns in bacterial isolates [27], particularly in resource-limited settings. It provides a cost-effective way to assess resistance and prior antibiotic exposure [28]. Together, these tools offer a comprehensive approach to AMR surveillance, guiding antibiotic stewardship, informing public health strategies, and improving local resistance monitoring. They support data-driven decisions to mitigate the spread of multidrug-resistant pathogens and enhance global AMR management efforts.

Antimicrobial resistance (AMR) is an escalating global health challenge that disproportionately impacts resource-limited regions, including the Caribbean. Although resistant bacterial strains, particularly *Escherichia coli*, are increasingly prevalent in both clinical and environmental settings, surveillance data from this region remain scarce. To address this critical gap, our study is the first to systematically compare phenotypic antibiotic resistance patterns of *E. coli* isolates from clinical urinary tract infections (UTIs) and municipal wastewater samples within a single community in Grenada.

By integrating data from these two sources, we provide a more comprehensive understanding of AMR dissemination pathways, bridging the clinical and environmental dimensions of resistance. This dual approach is especially valuable for resource-limited settings, where wastewater surveillance offers a cost-effective, community-level overview of AMR trends that can complement constrained clinical testing capacities.

By examining the similarities and differences between wastewater and clinical isolates, our study identifies priority antibiotics for stewardship programs and highlights the potential of wastewater-based surveillance as a scalable, practical tool for monitoring AMR in underserved regions like the Caribbean.

## 2. Materials and Methods

### 2.1. Samples

Samples were collected from January 2022 to October 2023 following the clinical laboratory calendar, including 31 wastewater samples and 34 antibiograms obtained from clinical UTI cases. All historical clinical UTI antibiogram data were de-identified, necessitating only minimal institutional review, which was granted. Although the sample size may seem small, it reflects the limited clinical and surveillance infrastructure in Grenada, a resource-constrained setting where systematic AMR monitoring has yet to be established. Wastewater samples were collected from strategic central locations designed to capture community-level bacterial exposure, while clinical isolates were obtained from all UTI cases during the same time period of time. This sample offers a foundational baseline for understanding resistance trends in both environmental and clinical settings in Grenada. While larger studies would improve generalizability, these findings are significant as they reveal parallels between wastewater and clinical resistance patterns, highlighting the potential of wastewater-based surveillance as a cost-effective and scalable approach to AMR monitoring. Future research involving extended sampling over time and across multiple regions is recommended to build on these insights.

### 2.2. Wastewater

Composite wastewater samples were collected using an ISCO autosampler (Lincoln, NE, USA, 68504), with 150 mL volumes sampled every 30 min. A total of 31 wastewater samples were collected from a central collection point within a community in Grenada, a resource-limited country in the Caribbean. The samples, spanning both the dry and wet seasons, were transported to the laboratory on ice at 4 °C to ensure sample integrity. An aliquot from each sample was used to detect the presence of *E. coli*, following protocols from a previous study [29]. In brief, 1 mL of raw wastewater was inoculated into 10 mL of lactose broth (LB) (Sigma-Aldrich, St. Louis, MO, USA) containing an inverted Durham tube and incubated at 37 °C for 24 h. Samples positive for lactose fermentation were selected for further analysis. These samples were then cultured on Eosin Methylene Blue (EMB) agar (Becton, Dickinson Company, Franklin Lakes, NJ, USA), and random metallic green colonies (50%) were streaked onto MacConkey (MAC) agar (Thermo Fisher Scientific, Waltham, MA, USA) and incubated for another 24 h at 37 °C. *E. coli* isolates from MAC agar were further cultured on Tryptic soy agar (Becton, Dickinson Company, Franklin Lakes, NJ, USA) for additional identification. Presumptive *E. coli* colonies were confirmed using the IMViC tests—indole, methyl red, Voges–Proskauer, and citrate (Becton, Dickinson Company, Franklin Lakes, NJ, USA) [30,31]. Final confirmation of *E. coli* was achieved using API 20E test strips (BioMérieux Inc., Durham, NC, USA) according to the manufacturing instructions.

### 2.3. Urine Samples

Urine cultures were processed following the Clinical Microbiology laboratory standard operation procedures. A clean-catch midstream urine sample was collected from patients presenting with UTI symptoms, ensuring minimal contamination, and sent to the Clinical Microbiology Lab for analysis. The urine was then inoculated onto blood agar (17501 W 98th St, Spc 30-60, Lenexa, KS 66219, USA) and MacConkey agar (Thermo Fisher Scientific. 168 Third Avenue. Waltham, MA USA) and incubated at 37 °C for 48 h. After incubation, colony counts were determined, and any significant growth (≥100,000 colony-forming units per milliliter of a single organism) was identified through biochemical testing. All the bacterial colonies were Gram-stained. All Gram-negative isolates were inoculated into API-20E (Analytical Profile Index, BioMetrieux Inc. Durham, NC, US) test strips and incubated at 37 °C for 24 h to confirm *E. coli.* Antimicrobial susceptibility testing was conducted to determine the sensitivity of isolated pathogens to commonly prescribed antibiotics. Antibiogram data were obtained for 34 *E. coli* isolates from urinary tract infections in this period of time

### 2.4. Susceptibility Testing

The antibiotic susceptibility phenotypes for both wastewater and clinical isolates were determined by the standard disk diffusion technique following the Clinical and Laboratory Standards Institute guidelines [32,33] against 11 different antibiotics (Table 1): amoxicillin/clavulanic acid (30 μg), ampicillin (10 μg), cefazolin (30 μg), cefoxitin (30 μg), ceftriaxone (30 μg), cefuroxime (30 μg), ciprofloxacin (5 μg), gentamicin (10 μg), nalidixic acid (30 μg), nitrofurantoin (300 μg), trimethoprim-sulfamethoxazole (1.25 μg, 23.75 μg). Only wastewater isolates were subjected to Aztreonam (30 μg) (Becton, Dickinson and Company) [34,35]. These antibiotics were chosen because they are routinely used by the clinical microbiology laboratory for testing urinary tract infection (UTI) isolates from clinical samples. Aztreonam (30 μg) was tested exclusively on wastewater isolates, as it is not commonly used in the clinical assessment of suspected UTI cases. Additionally, aztreonam is classified as a Reserved Group antibiotic, requiring intravenous or intramuscular administration for UTI patients and no data from the clinical samples were available.

The inhibition zones were measured, and isolates were classified as resistant intermediate or susceptible according to CLSI ranges [33]. Quality control checks were performed using *E. coli* strain ATCC 25922. Multi-drug resistant (MDR) isolates were selected among those that had resistance to 3 or more of the antibiotics tested [36].

### 2.5. Data Analysis

The MAR index was calculated by the following formula: a/b (a = the number of antibiotics the isolate was resistant to, and b = the total number of antibiotics the isolate was exposed to). A bacterial isolate having an MAR index ≥ 0.2 would have originated from a high-risk source of contamination where multiple antibiotics are used [37,38,39]. The maximum possible MAR value is 1.00, obtained when all isolates are resistant to all antibiotics it is tested against [40]. The antibiogram data were analyzed utilizing the CIA list [41] and WHO AWaRe Antibiotic guidelines (Figure 1) [26].

## 3. Results

### 3.1. Antibiotic Resistance Patterns Based on the CIA List 

This study assessed 34 *E. coli* isolates from clinical UTI samples and 86 *E coli* isolates from 31 samples of municipal wastewater. In total, 18 of the 34 (52.9%) isolates from clinical UTI samples were resistant to at least one of the antibiotics tested, while 13 of 86 (15.1%) municipal wastewater isolates were resistant to at least one of the antibiotics tested. Using the CIA list [41], similar patterns were found for wastewater and clinical samples. Ampicillin showed the highest resistance levels for the Critically Important group, followed by amoxicillin/clavulanic acid and nalidixic acid. In the Highly Important group, both wastewater and clinical samples showed resistance for sulfamethoxazole-trimethoprim, and for the Important group, nitrofurantoin showed resistance in both wastewater and clinical samples (Table 2 and Table 3). Similar patterns were observed with all antibiotics tested except ceftriaxone and ciprofloxacin, with no resistant isolates in the wastewater.

**Figure 2:**
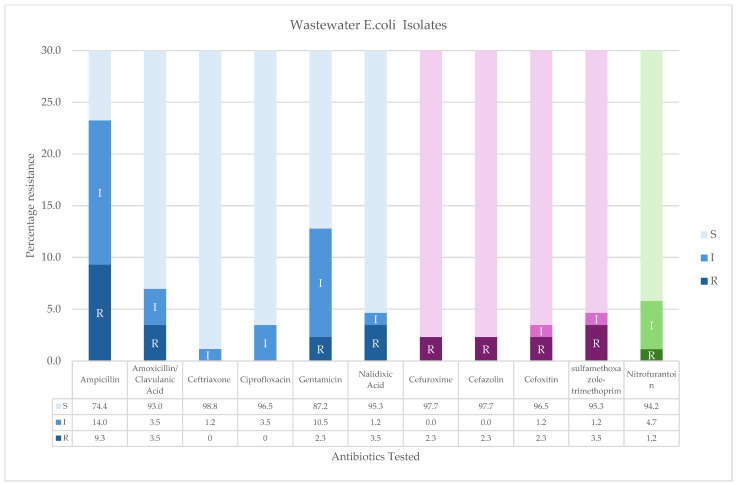
Susceptibility patterns of *E. coli* strains to WHO Medically Important Antimicrobials for Human Medicine rom wastewater: (BLUE) Critically Important antimicrobials, (PURPLE) Highly Important, and (Green) Important to human medicine. The WHO CIA list used was as a reference to help formulate and prioritize risk assessment and risk management strategies for containing antimicrobial resistance: (A) Critically Important: Antibiotics that meet both criteria: (1) they are the sole or limited therapeutic option for treating life-threatening infections, and (2) they are used to treat infections caused by bacteria from non-human sources or carry resistance genes originating from non-human sources. (B) Highly Important: Antibiotics that meet only one of the following criteria: (1) they are the sole or limited treatment option for life-threatening infections, or (2) they are used for infections caused by bacteria from non-human sources or with resistance genes from non-human sources. (C) Important: Antimicrobial classes used in humans that do not meet criteria (1) or (2) [41].

The antibiogram data for the municipal wastewater *E. coli* isolates were analyzed based on their susceptibility patterns to The CIA list (Critically important, Highly Important, and Important) [41]. Critically Important antimicrobials with resistance were (Figure 2): ampicillin (9.3%), amoxicillin/clavulanic acid (3.5%), nalidixic acid (3.5%), and gentamicin (2.3%). All the isolates were susceptible to ceftriaxone and ciprofloxacin. For antibiotics categorized as Highly Important, resistance was observed to sulfamethoxazole-trimethoprim (3.5%) cefazolin (2.3%), cefoxitin (2.3%), and cefuroxime(2.3%). Nitrofurantoin (1.2%) was the lone antibiotic in the Important category (Figure 2).

**Figure 3:**
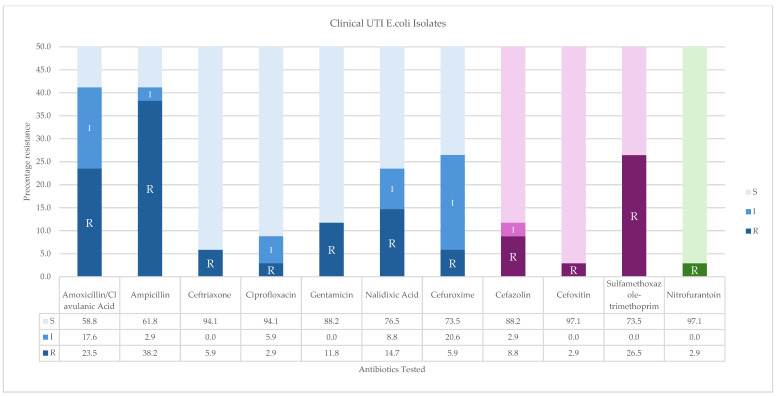
Susceptibility pattern of *E. coli* strains to WHO Medically Important Antimicrobials for Human Medicine from clinical samples: (BLUE) Critically Important antimicrobials, (PURPLE) Highly Important, and (Green) Important to human medicine.

The antibiogram data for the Clinical UTI *E. coli* isolates were also analyzed based on their susceptibility patterns to the CIA list [41] (Figure 3). For the category of Critically Important, the following patterns were found: ampicillin (38.2%), amoxicillin/clavulanic acid (23.5%), nalidixic acid (14.7%), and gentamicin (11.8%). Less than 10% of the strains showed resistance to ceftriaxone (5.9%) and ciprofloxacin (2.9%). For antibiotics categorized as Highly Important, resistance was observed to sulfamethoxazole-trimethoprim (26.5%) and less than 10% for cefazolin (8.8%), cefoxitin (2.9%), and cefuroxime(5.9%). Nitrofurantoin (2.9%) was the lone antibiotic in the Important category (Figure 3).

### 3.2. Antibiotic Resistance Based on ACCESS, WATCH, and RESERVE (‘AWaRe’) Classification 

**Figure 4:**
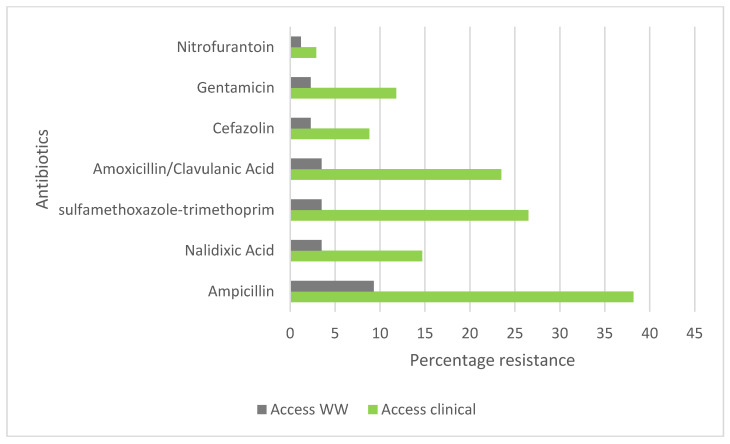
WHO AWaRe ACCESS classification for *E. coli* isolated from wastewater and clinical urinary tract isolates. The classification is based, in part, on the risk of developing antibiotic resistance and their importance to medicine. The ACCESS category of this classification includes empiric first or second choice for treatment of most common infection and generally available [42].

**Figure 5:**
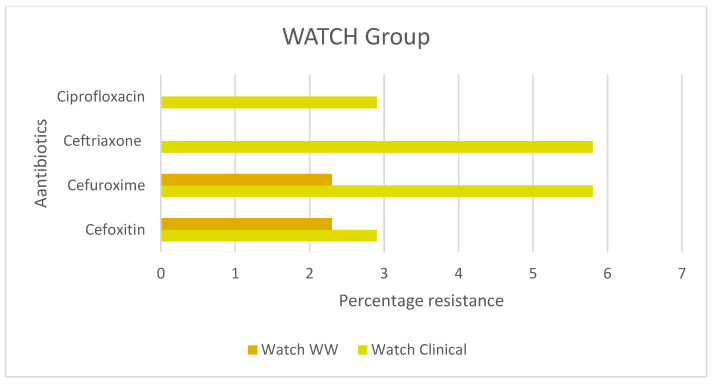
WHO AWaRe: WATCH classification for *E. coli* isolated from wastewater and clinical urinary tract isolates. AWaRe classification: The classification is based, in part, on the risk of developing antibiotic resistance and their importance to medicine. WATCH category: Antibiotics that have higher toxicity issues and higher potential to negatively impact AMR. They should only be prescribed for specific infections [42].

Based on the ‘AWaRe’ classification of antibiotics (Figure 1) [25], the highest resistance was observed to antibiotics from the AWaRe-ACCESS group (Figure 4), ampicillin 9.3% in wastewater and 38.2% in clinical UTI isolates, and the lowest resistance was observed to nitrofurantoin 1.2% of wastewater isolates and 2.9% of the clinical UTI isolates. The remaining antibiotic resistance varied between 2% and 10%. In the AWaRe-WATCH group (Figure 5), similar resistance patterns were found for cefuroxime and cefoxitin but not ciprofloxacin and ceftriaxone in the wastewater (Figure 4). Aztreonam, a monobactam AWaRe-RESERVE antibiotic, was tested only in wastewater since it is not recommended by the WHO AWaRe classification for routine treatment of UTIs, showing sensitivity in 100% of the isolates.

### 3.3. Multidrug Resistance Patterns (MDR)

A total of nine multidrug resistance (MDR) patterns were observed across all isolates (Table 4), three isolates from wastewater and seven from clinical UTI samples. The most common MDR patterns found common to both clinical and wastewater isolates were AMC/AM (eight out of nine isolates), AMC/AM/CZ (five out of nine isolates), AMC/AM/CZ/CXM (four out of nine isolates), and AMC/AM/CZ/CXM/FOX (two out of nine isolates).

### 3.4. Multiple Antibiotic Resistance Index (MAR)

The MAR index for *E. coli* isolates from wastewater is presented in Table 5, while the clinical *E. coli* isolates are presented in Table 6.

## 4. Discussion

This study examined the phenotypic antibiotic resistance patterns of *E. coli* isolates from municipal wastewater and clinical UTI samples within a community in Grenada. A total of 120 isolates were analyzed, including 86 from 31 wastewater samples and 34 from clinical UTI cases. Of the clinical UTI isolates, 18 out of 34 (52.9%) were resistant to at least one antibiotic, while 13 out of 86 (15.1%) wastewater isolates showed resistance to at least one antibiotic. Other studies have also found an increase in resistance closer to hospital sources. For example, Kwak et al. [40] found that in Stockholm, 34% of *E. coli* isolates from urban wastewater and 55% from hospital wastewater were resistant to at least one of the 10 tested antibiotics.

This study found that *E. coli* was most resistant to ampicillin (9.3% in wastewater and 38.2% in clinical samples) and also found significantly low resistance for nitrofurantoin (1.2% in wastewater and 2.9% in clinical samples), consistent with other studies. In Zambia, a developing country where irrational prescribing of antibiotics is prevalent [43], Kasanga et al. [44] found a high resistance of *E. coli* to some antibiotics that are commonly used in humans. Out of 450 samples (300 clinical samples and 150 environmental samples), 81.4% of the isolates were highly resistant to ampicillin but highly susceptible to nitrofurantoin (89.3%). In Norwegian wastewater with low consumption of antibiotics in both the human and veterinary medical sectors, relatively low prevalence of antibiotic resistant fecal coliforms and enterococci were found compared to that of other, southern European countries regarded as high consumers of antibiotics [45]. In Sweden, Paulshus et al. [46] found that effluent resistance from hospital and non-hospital wastewater to at least one of the 9 antibiotics was observed in 45% of the hospital wastewater isolates, and 33 to 44% to non-hospital wastewater. Resistance to ampicillin was the most common (31%).

Ampicillin is a safe and effective antibiotic, widely used in pregnancy and pediatrics and available in both oral and injectable forms [47]. Its frequent use in treating various infections, however, has contributed to growing resistance, complicating treatment and underscoring the need for prudent antibiotic stewardship to preserve its efficacy [47]. Interestingly, high rates of resistance to ampicillin have been reported even in regions with strict antibiotic regulations, such as Sweden [46], where increased resistance rates suggest other contributing factors beyond overuse. In our study, ampicillin resistance was found in all multidrug-resistant *E. coli* isolates, reflecting *E. coli*’s remarkable ability to acquire resistance genes—largely through horizontal gene transfer. This adaptability makes *E. coli* multidrug resistance a significant and expanding challenge in both human and veterinary health.

The MAR index has been used in studies from low- and middle-income countries as an alternative method of tracking the source and predicting previous exposure to antibiotics to bacteria [28]. Isolates with a MAR index of greater than 0.2 would have originated from a high-risk source of contamination where multiple antibiotics are used [37,38,39]. The observed MAR classification of *E. coli* isolates, with 7% of wastewater samples and 38% of clinical samples showing MAR indexes greater than 0.2, aligns with global concerns about the spread of antibiotic resistance in environmental settings. This highlights the growing challenge of resistance in wastewater, underscoring the importance of continued surveillance, careful antibiotic use, and the development of effective strategies to address the public health risks associated with resistant strains. Such findings stress the need for robust environmental monitoring and timely interventions to curb the further spread of multidrug-resistant organisms, particularly in areas with intensive human activity and waste management practices.

The present study also found significantly low resistance for nitrofurantoin (1.2% in wastewater, and 2.9% in clinical samples). In a European study that spanned 20 centers in Belgium, the UK, Italy, Spain, and Russia, similar findings were reported with resistance rates of 1.5% to nitrofurantoin when tested in UTI isolates [48]. Another study conducted in Gothenburg, Sweden, found that 0.9% of *E. coli* isolates from hospital wastewater had resistance to nitrofurantoin, but no resistance was detected in municipal sewage isolates [49]. Research conducted on the Caribbean Island of Curaçao found that urinary tract infections with *E. coli* had low resistance to nitrofurantoin, with resistance levels below 4% [50], which is similar to our findings for clinical UTI 2.9% for nitrofurantoin.

This indicates that nitrofurantoin (an ACCESS group drug) would still be effective in treating uncomplicated UTIs in the studied population [51].

Previous studies conducted by Sharma et al. [52] using retrospective data of all clinically diagnosed infections of UTI for the years 2015 through 2017 (3867 urine samples) from the bacteriology laboratory of the General Hospital, Grenada W.I highlighted a varying degree of resistance to antimicrobial drugs in *E. coli* and other causes of UTI. They showed a pattern of high resistance to ampicillin, Bactrim, and Augmentin. Since the recommended first-line treatment for a UTI infection in Grenada is trimethoprim/sulfamethoxazole (Bactrim) followed by ciprofloxacin and amoxicillin/clavulanate acid (Augmentin) [52], they argue that the resistance pattern observed in their study could be due to the past promoted use of ciprofloxacin and the current recommended use of Bactrim and Augmentin for most UTI infections. Trimethoprim/sulfamethoxazole is a Highly Important and AWaRe-ACCESS drug found in our study to have a 26% resistance in the clinical samples, and 3.5% in wastewater. Amoxicillin/clavulanate acid is a Critically Important and AWaRe-ACCESS drug found in our study to have 23.5% resistance in the clinical samples and 3.5% in wastewater. Ciprofloxacin is a Critically Important and AWaRe-WATCH drug with 2.9% resistance in clinical samples and 0% in wastewater, as found in our study. Analyzing our data in light of the WHO CIA list and AWaRe classifications, we found that the best choice for antibiotic recommendations in the groups with low resistance in the Important category, meaning antibiotics commonly used for treating infections where there are more treatment options available and where their use poses a lower immediate risk and had AWaRe-ACCESS classification meaning that is an empiric first or second choice for treatment of most common infection and should be generally available. The antibiotic that fit these characteristics was nitrofurantoin. Research conducted by [53] also supports the use of nitrofurantoin as an appropriate alternative to other drugs, such as Fosfomycin, for the treatment of uncomplicated UTIs.

This study offers valuable insights into antimicrobial resistance (AMR) patterns in *E. coli* isolates from Grenada. However, the limited availability of regional data for direct comparison underscores the challenges of contextualizing our findings within the Caribbean. Despite this limitation, our results align with global trends observed in other resource-limited settings, where similar resistance patterns to ampicillin, sulfamethoxazole-trimethoprim, and other first-line antibiotics have been reported. These findings highlight the urgent need to expand AMR surveillance efforts across the Caribbean to establish regional benchmarks and monitor resistance trends over time.

This study concentrated on phenotypic antibiotic resistance patterns in *E. coli* isolates from wastewater and clinical UTI samples, without investigating the underlying genetic mechanisms, such as the presence of resistance genes or horizontal gene transfer. While understanding these mechanisms is crucial for identifying the pathways driving AMR dissemination, it was beyond the scope of this paper. In resource-limited settings like ours, surveillance systems are more feasible to implement using phenotypic data rather than genotypic data. Future research incorporating molecular techniques, such as whole-genome sequencing or PCR-based analysis, could provide deeper insights into the genetic factors contributing to resistance in this community. To the best of our knowledge, this study is the first to assess antibiotic resistance in *E. coli* isolates from untreated municipal wastewater samples and clinical samples from the population contributing to the wastewater collected for that same time period on the island of Grenada. These studies highlight how wastewater can be used as a complementary tool to help monitor phenotypic antibiotic resistance.

## 5. Conclusions

Antimicrobial resistance (AMR) is a growing global health threat, with the rise of multidrug-resistant infections and superbugs posing serious challenges. A critical strategy to combat AMR is implementing strong antimicrobial stewardship and management practices. Our study suggests that phenotypic antibiotic resistance data from municipal wastewater samples closely reflects the antibiotic resistance patterns observed within the studied population over the same period. However, further refinement and longer observation periods are necessary to improve the method’s predictive accuracy for clinical resistance in the population, which could support expanding this pilot study to larger settings.

Grenada currently lacks robust monitoring capacity for AMR patterns. We propose using wastewater as a supplemental method for tracking AMR, providing essential data on *E. coli* resistance patterns and potentially guiding treatment options. This approach can support AMR surveillance until a more comprehensive monitoring system is developed, and it has the potential for expansion to monitor resistance in other bacterial pathogens as well.

## Figures and Tables

**Table 1:** Antibiotics used in this study for susceptibility testing.

Antibiotic	Abbreviation/ Concentration µg	Class of Antibiotic
Amoxicillin/Clavulanic Acid	AMC 30	Penicillin + beta-lactamase inhibitor
Ampicillin	AM 10	Penicillin (Beta-lactam)
Aztreonam *	ATM 30	Monobactam
Cefazolin	CZ 30	First generation cephalosporin
Cefoxitin	FOX 30	Second generation cephalosporin
Ceftriaxone	CRO 30	Third-generation cephalosporin
Cefuroxime	CXM 30	Second generation cephalosporin
Ciprofloxacin	CIP 5	Fluoroquinolone
Gentamicin	GM 10	Aminoglycosides
Nalidixic Acid	NA30	Quinolones
Nitrofurantoin	FM 300	Nitrofuran-derivative
Sulfamethoxazole-trimethoprim	SXT25	Folate pathway antagonist

* Aztreonam was only assessed for wastewater *E. coli* isolates.

**Table 2:** Patterns of resistance, determined by disk diffusion, for *E. coli* isolates from wastewater.

Antibiotic	R **		I **		S **	
	N *	% *	N *	% *	N *	% *
Amoxicillin/Clavulanic Acid	3	3.5	3	3.5	80	93.0
Ampicillin	8	9.3	12	14.0	66	76.7
Aztreonam	0	0	0	0.0	86	100.0
Cefazolin	2	2.3	0	0.0	84	97.7
Cefoxitin	2	2.3	1	1.2	83	96.5
Ceftriaxone	0	0	1	1.2	85	98.8
Cefuroxime	2	2.3	0	0.0	84	97.7
Ciprofloxacin	0	0	3	3.5	83	96.5
Gentamicin	2	2.3	9	10.5	75	87.2
Nalidixic Acid	3	3.5	1	1.2	82	95.3
Nitrofurantoin	1	1.2	4	4.7	81	94.2
sulfamethoxazole-trimethoprim	3	3.5	1	1.2	82	95.3

* N: number of isolates, (% percentage); ** Terms: Sensitive/Susceptible (S): A microorganism is classified as susceptible when standard dosing is likely to be clinically effective in treating the infection. Intermediate (I): A microorganism is considered intermediate if it may respond to higher drug exposure, either through increased dosage or extended exposure time, potentially resulting in successful treatment. Resistant (R): A microorganism is resistant if it continues to grow despite normal drug dosage, making treatment unreliable [41].

**Table 3:** Patterns of resistance, determined by disk diffusion, for *E. coli* isolates from clinical UTI samples.

Antibiotic	R **		I **		S **	
	N *	% *	N *	%*	N *	% *
Amoxicillin/Clavulanic Acid	8	23.5	6	17.6	20	58.8
Ampicillin	13	38.2	1	2.9	21	61.8
Cefazolin	3	8.8	1	2.9	30	88.2
Cefoxitin	1	2.9	0	0	33	97.1
Ceftriaxone	2	5.9	0	0	32	94.1
Cefuroxime	2	5.9	7	20.6	25	73.5
Ciprofloxacin	1	2.9	2	5.9	32	94.1
Gentamicin	4	11.8	0	0	30	88.2
Nalidixic Acid	5	14.	3	8.8	26	76.5
Nitrofurantoin	1	2.9	0	0	33	97.1
Sulfamethoxazole-trimethoprim	9	26.5	0	0	25	73.5

* N: number of isolates, (% percentage); ** Terms: Sensitive/Susceptible (S): A microorganism is classified as susceptible when standard dosing is likely to be clinically effective in treating the infection. Intermediate (I): A microorganism is considered intermediate if it may respond to higher drug exposure, either through increased dosage or extended exposure time, potentially resulting in successful treatment. Resistant (R): A microorganism is resistant if it continues to grow despite normal drug dosage, making treatment unreliable [41].

**Table 4:** Phenotypic antibiotic resistance profile of *E. coli* isolates from wastewater and clinical UTI samples.

Pattern	Number of Isolates	Source	Phenotypic Resistance Profile
A	2	Clinical UTI	AMC AM SXT
B	1	Clinical UTI	AM GM SXT
C	1	Wastewater	AMC AM FOX
D	1	Wastewater	AMC AM CZ CXM NA
E	1	Wastewater	AMC AM CZ CXM FOX
F	1	Clinical UTI	AMC AM CZ CXM FOX CRO GM
E	1	Clinical UTI	AMC AM CZ GM SXT CIP NA
F	1	Clinical UTI	AMC AM CZ CXM CRO GM SXT

Key: Amoxicillin/clavulanic acid (AMC), ampicillin (AM), cefazolin (CZ), cefuroxime (CXM), gentamicin (GM), cefoxitin (FOX), nalidixic acid (NA), ciprofloxacin (CIP), sulfamethoxazole-trimethoprim (SXT), ceftriaxone (CRO), nitrofurantoin (FM).

**Table 5:** MAR index of *E. coli* isolates from wastewater samples.

Number of Isolates *N* = 86 (100%)	MAR
73 (84.9)	0.0
7 (8.1)	0.1
3 (3.5)	0.2
1 (1.2)	0.3
2 (2.3)	0.4
TOTAL: 6 (7)	>0.2

Total value, on grey background, refers to bacterial isolates: with MAR index ≥ 0.2 (originated from a high-risk source of contamination).

**Table 6:** MAR index of *E. coli* isolates from clinical UTI.

Number of Isolates *N* = 34 (100%)	MAR
16 (47.1)	0.0
5 (14.7)	0.1
7 (20.6)	0.2
2 (5.9)	0.3
1 (2.9)	0.4
0	0.5
3 (8.8)	0.6
TOTAL: 13 (38.2)	>0.2

Total value, on grey background, refers to bacterial isolates: with MAR index ≥ 0.2 (originated from a high-risk source of contamination).

## Data Availability

All data are provided in the article.
